# Effects of Cadmium, Thallium, and Vanadium on Photosynthetic Parameters of Three Chili Pepper (*Capsicum annuum* L.) Varieties

**DOI:** 10.3390/plants12203563

**Published:** 2023-10-13

**Authors:** María de la Luz Buendía-Valverde, Fernando C. Gómez-Merino, Tarsicio Corona-Torres, Rodrigo Aníbal Mateos-Nava, Libia I. Trejo-Téllez

**Affiliations:** 1 Laboratory of Plant Nutrition, Department of Soil Science, College of Postgraduates in Agricultural Sciences, Campus Montecillo, Montecillo, Texcoco 56264, Mexico; buendia.luz@colpos.mx; 2 Department of Genetic Resources and Productivity-Plant Physiology, College of Postgraduates in Agricultural Sciences, Campus Montecillo, Montecillo, Texcoco 56264, Mexico; fernandg@colpos.mx; 3 Laboratory of Plant Tissue Culture, Department of Sustainable Agri-Food Innovation, Collaborative Research Group at College of Postgraduates in Agricultural Sciences, Campus Córdoba, Manuel León, Amatlán de los Reyes 94953, Mexico; 4 Department of Genetic Resources and Productivity-Genetics, College of Postgraduates in Agricultural Sciences, Campus Montecillo, Montecillo, Texcoco 56264, Mexico; tcoronat@colpos.mx; 5 Research Unit in Genetics and Environmental Toxicology (UIGTA), Multidisciplinary Experimental Research Unit (UMIE-ZAP 9-020), L5 PA Laboratory, Faculty of Higher Studies-Zaragoza, National Autonomous University of Mexico, Campus II, Mexico City 15000, Mexico; a_mateos_n@yahoo.com.mx

**Keywords:** non-essential metals, hormetic responses, internal CO_2_ concentration, net photosynthetic rate, stomatal conductance

## Abstract

Photosynthesis is a crucial process supporting life on Earth. However, unfavorable environmental conditions including toxic metals may limit the photosynthetic efficiency of plants, and the responses to those challenges may vary among genotypes. In this study, we evaluated photosynthetic parameters of the chili pepper varieties Jalapeño, Poblano, and Serrano exposed to Cd (0, 5, 10 µM), Tl (0, 6, 12 nM), and V (0, 0.75, 1.5 µM). Metals were added to the nutrient solution for 60 days. Stomatal conductance (*Gs*), transpiration rate (*Tr*), net photosynthetic rate (*Pn*), intercellular CO_2_ concentration (*Ci*), instantaneous carboxylation efficiency (*Pn*/*Ci*), instantaneous water use efficiency (*instWUE*), and intrinsic water use efficiency (*iWUE*) were recorded. Mean *Pn* increased with 12 nM Tl in Serrano and with 0.75 µM V in Poblano. Tl and V increased mean *Tr* in all three cultivars, while Cd reduced it in Jalapeño and Serrano. *Gs* was reduced in Jalapeño and Poblano with 5 µM Cd, and 0.75 µM V increased it in Serrano. *Ci* increased in Poblano with 6 nM Tl, while 12 nM Tl reduced it in Serrano. Mean *instWUE* increased in Poblano with 10 µM Cd and 0.75 µM V, and in Serrano with 12 nM Tl, while 6 nM Tl reduced it in Poblano and Serrano. Mean *iWUE* increased in Jalapeño and Poblano with 5 µM Cd, in Serrano with 12 nM Tl, and in Jalapeño with 1.5 µM V; it was reduced with 6 nM Tl in Poblano and Serrano. *Pn*/*Ci* increased in Serrano with 5 µM Cd, in Jalapeño with 6 nM Tl, and in Poblano with 0.75 µM V. Interestingly, Tl stimulated six and inhibited five of the seven photosynthetic variables measured, while Cd enhanced three and decreased two variables, and V stimulated five variables, with none inhibited, all as compared to the respective controls. We conclude that Cd, Tl, and V may inhibit or stimulate photosynthetic parameters depending on the genotype and the doses applied.

## 1. Introduction

To complete their life cycle, higher plants require essential elements classified as macronutrients and micronutrients. Macronutrients (N, P, K, Ca, Mg, and S) are required in greater quantities and normally constitute 0.1% or more of plant weight on a dry basis, while micronutrients (Cl, Fe, B, Mn, Cu, Zn, Mo, and Ni) are required in lower concentrations and constitute less than 0.1% of the plant weight on a dry basis [1]. Among these essential elements, N and Mg play a pivotal role in photosynthesis, since the chlorophyll molecule consists of a central Mg atom surrounded by a N-containing structure called a porphyrin ring. During the absorption process of essential elements, plants can also absorb other non-essential and potentially toxic elements such as As, Cd, Cr, Pb, Tl, and V. Depending on the levels absorbed by plants, these elements may alter physiological and metabolic processes, and eventually accumulate at different trophic levels and contaminate the food chain [2]. Trophic transfer, bioaccumulation, and biomagnification of hazardous metals in food chains had toxic implications for human health. Indeed, organisms at higher trophic levels in food chains are more vulnerable to biomagnification [3,4].

According to the Toxic Release Inventory (TRI) of the United States Environmental Protection Agency (EPA), elements such as Cd, Tl, and V represent a threat to human health and to the environment [5]. Although these elements are found at low concentrations in nature, in recent decades industrial waste, vehicle emissions, and extensive cultivation methods implemented in agriculture have increased their release into the environment, which has become categorized as a high-risk factor in soil-plant systems [5,6,7].

In the soil solution, non-essential metals can be found mixed with organic and inorganic materials, competing with essential elements for root absorption sites. Although their mobility and availability are limited by different soil properties, non-essential metals can be very competitive and dynamic in the absorption process in root cells [8,9].

Cereals such as wheat (*Triticum aestuvum* L.), rice (*Oryza sativa* L.), rye [*Secale cereale* (L.) M.Bieb.], oats (*Avena sativa* L.), and millet (*Panicum miliaceum* L.), as well as tubers, fruits, vegetables, and sugarcane (*Saccharum* spp.) show toxic symptoms when exposed to high levels of some non-essential metals (i.e., Cd, Cr, Hg and Pb), which, when consumed by humans, represent a threat to public health [10,11].

In plants, high levels of non-essential metals cause alterations at the cellular, molecular, and morphological levels, triggering enzyme inactivation, blocking functional groups, displacing or substituting essential elements and disrupting photosynthesis [12,13].

Photosynthesis is the process of converting solar energy into chemical energy and is essential for the optimal growth and development of plants. Under abiotic stress conditions imposed by toxic levels of non-essential metals, photosynthesis may be altered through gas exchange imbalance, which is regulated mainly by the opening and closing of stomata [14]. High levels of non-essential metals can further cause chlorophyll degradation, as well as low water absorption and low CO_2_ fixation [15,16]. In the unicellular green alga *Micrasterias denticulata*, the application of 5 to 150 µM Cd negatively affected the transport of electrons in photosystem II [13]. Furthermore, Cd modifies the structure of chloroplasts, the functionality of the thylakoid membranes and the antenna complex, which negatively affects photosynthetic activity, gas exchange, and water absorption [17,18]. Under Cd stress conditions, photochemical efficiency, chlorophyll content, and photosynthetic intensity are significantly reduced, because this metal damages the light-harvesting complex and photosystems I and II [19,20]. In white mustard (*Sinapis alba* L.), toxic levels of Tl (i.e., 100 and 1000 µg L^−1^) decreased the photochemical activity of the reaction centers of photosystems I and II, causing oxidation of pigments in leaves, smaller chloroplasts, decrease and disorder of grana [21]. In tomato, the application of 40 mg L^−1^ V reduced gas exchange parameters and decreased the biosynthesis of photosynthetic pigments [22]. Nonetheless, these metals may also result in beneficial responses in plants, in a hormetic manner.

Since the 1990s, the phenomenon of hormesis has regained importance in biological sciences, and non-essential metals have played a very important role in the study of this phenomenon. When living beings are exposed to non-essential metals, it is possible to observe biphasic response curves, in which negative or inhibitory responses can be observed at high concentrations of the metal, while at low concentrations it is possible to observe positive or stimulating responses, depending on the adaptive and resistance capacity of the organism, represented by its biological plasticity [23,24]. The definition of the hormetic curves in response to exposure to non-essential metals provides relevant information to determine the tolerance levels of plants and their impacts on the vital process of photosynthesis [25]. Specifically, the effect of the application of Cd, Tl, and V on photosynthetic parameters of different genotypes of chili pepper (*Capsicum annuum* L.) has been little investigated. Exploring those crucial physiological responses will expand our understanding and surely lay the groundwork for the regulation of stress tolerance mechanisms, wider adaptability, higher survival rate, and yield potential of different plant species grown under environments contaminated with hazardous metals. The objective of this study was to evaluate different photosynthetic variables (i.e., stomatal conductance [*Gs*], transpiration rate [*Tr*], net photosynthetic rate [*Pn*], intercellular CO_2_ concentration [*Ci*], instantaneous carboxylation efficiency [*Pn*/*Ci*], instantaneous water use efficiency [*instWUE*], and intrinsic water use efficiency [*iWUE*]) of three hydroponically-grown chili pepper varieties, namely Jalapeño, Poblano, and Serrano, in response to treatment with the non-essential metals Cd, Tl, and V.

## 2. Results

### 2.1. Net Photosynthetic Rate (Pn)

**Figure 1:**
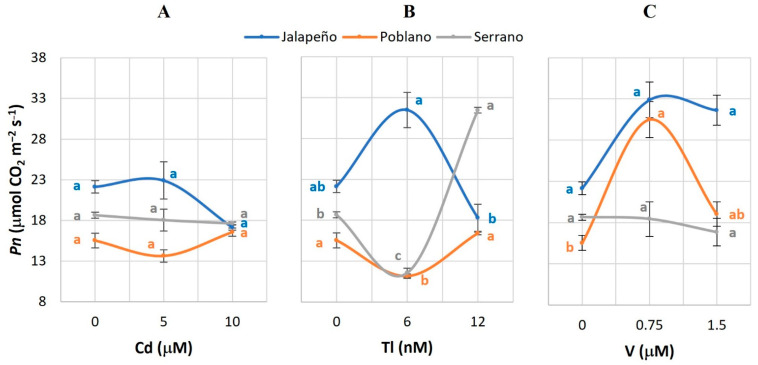
Net photosynthetic rate (*Pn*) in three chili pepper varieties (Jalapeño, Poblano, and Serrano), treated with different doses of cadmium (**A**), thallium (**B**) or vanadium (**C**), for 60 d in a hydroponic system. Means ± SEM. Different letters in each subfigure and variety indicate significant statistical differences among treatments (Tukey, *p* ≤ 0.05). *n* = 4.

The application of Tl and V had significant effects on *Pn* (Figure 1). The effects of Tl in Serrano chili pepper stand out: the 6 nM Tl dose reduced the *Pn* value by 38.1%; when applying 12 nM Tl, this variable increased 172.5%, in both cases with respect to the control (Figure 1B). The application of 0.75 µM V increased the *Pn* by 141.6% in Poblano, with respect to the control (Figure 1C).

### 2.2. Transpiration Rate (Tr)

**Figure 2:**
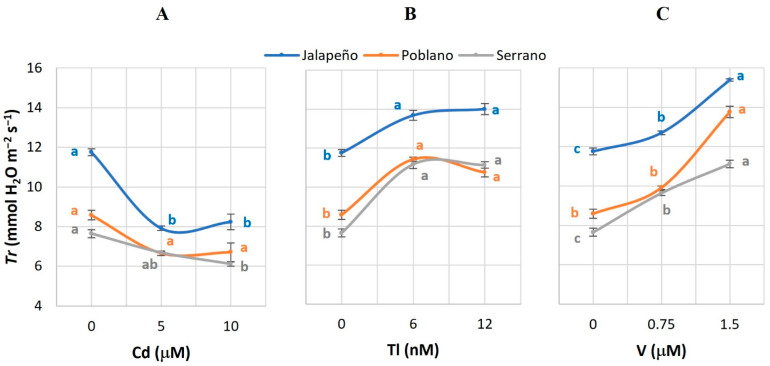
Transpiration rate (*Tr*) in three chili pepper varieties (Jalapeño, Poblano, and Serrano), treated with different doses of cadmium (**A**), thallium (**B**) or vanadium (**C**), for 60 d in a hydroponic system. Means ± SEM. Different letters in each subfigure and variety indicate significant statistical differences among treatments (Tukey, *p* ≤ 0.05). *n* = 4.

In Jalapeño, the application of 10 µM Cd reduced *Tr* by 29.9%, and in Serrano by 20%, compared to the control. When applying 5 µM Cd, this variable was reduced by 32.6% in Jalapeño peppers, compared to the control (Figure 2A). Tl application at both doses significantly increased *Tr* in all three chili pepper varieties, with average increases of 17.8, 29.3, and 45.9% in Jalapeño, Poblano, and Serrano, respectively, compared to the control (Figure 2B). In the three evaluated chili pepper varieties, the application of V increased *Tr*. When applying 0.75 and 1.5 µM V, the value of *Tr* in Jalapeño increased by 8.1 and 30.9%, respectively, in Poblano by 15.5 and 60.4%, and in Serrano by 26 and 45.4%; in all cases with respect to the controls (Figure 2C).

### 2.3. Stomatal Conductance (Gs)

**Figure 3:**
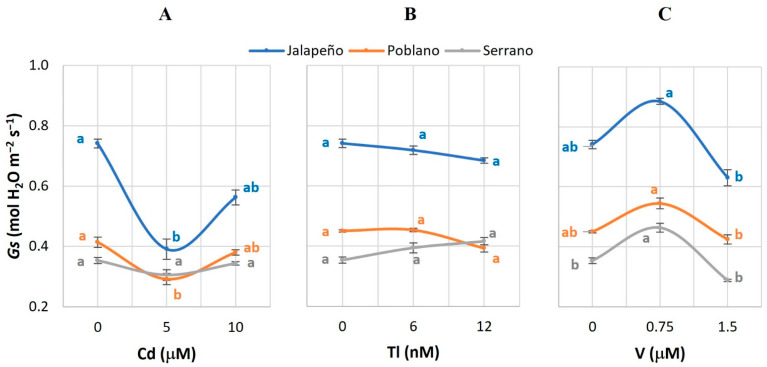
Stomatal conductance (*Gs*) in three chili pepper varieties (Jalapeño, Poblano, and Serrano), treated with different doses of cadmium (**A**), thallium (**B**) or vanadium (**C**), for 60 d in a hydroponic system. Means ± SEM. Different letters in each subfigure and variety indicate significant statistical differences among treatments (Tukey, *p* ≤ 0.05). *n* = 4.

The medium dose of Cd (5 µM) reduced *Gs* by 47.2 and 29.5% in Jalapeño and Poblano, compared to the controls (Figure 3A). The evaluated doses of Tl did not affect this variable (Figure 3B), while the application of 0.75 µM V increased *Gs* by 31% in Serrano. Although this same trend was observed in Jalapeño and Poblano, V did not have statistically significant effects on this variable (Figure 3C).

### 2.4. Intercellular CO_2_ Concentration (Ci) in the Leaf

**Figure 4:**
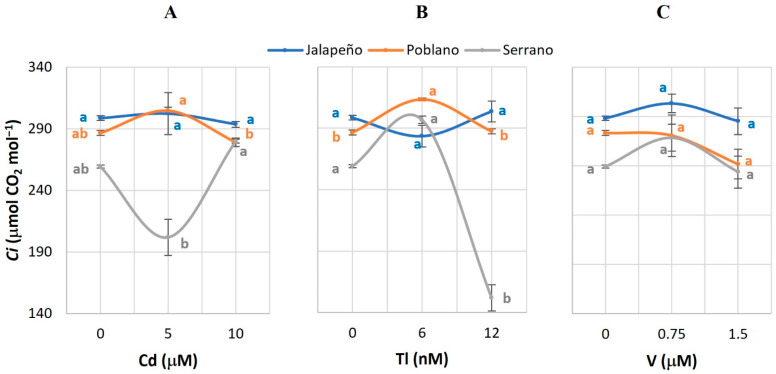
Intercellular CO_2_ concentration (*Ci*) in three chili pepper varieties (Jalapeño, Poblano, and Serrano), treated with different doses of cadmium (**A**), thallium (**B**) or vanadium (**C**), for 60 d in a hydroponic system. Means ± SEM. Different letters in each subfigure and variety indicate significant statistical differences among treatments (Tukey, *p* ≤ 0.05). *n* = 4.

The application of Cd or V did not cause differences in the *Ci* of the chili pepper varieties with respect to the controls (Figure 4A,C). In Serrano, the 12 nM Tl treatment reduced *Ci* by 41.3% compared to the control. In Poblano, the application of 6 nM Tl increased *Ci* by 9.4% compared to the control (Figure 4B).

### 2.5. Instantaneous Water Use Efficiency (instWUE)

**Figure 5:**
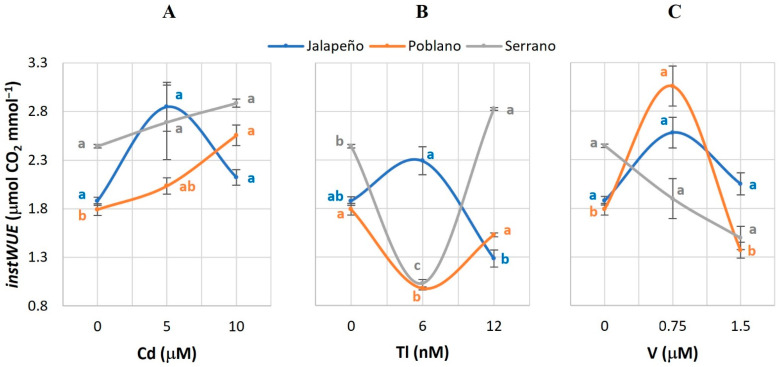
Instant water use efficiency (*instWUE*) in three chili pepper varieties (Jalapeño, Poblano, and Serrano), treated with different doses of cadmium (**A**), thallium (**B**) or vanadium (**C**), for 60 d in a hydroponic system. Means ± SEM. Different letters in each subfigure and variety indicate significant statistical differences among treatments (Tukey, *p* ≤ 0.05). *n* = 4.

The application of Cd increased the values of *instWUE* in the Poblano and Serrano varieties, although significant differences were only observed in Poblano with respect to the control when applying 10 µM Cd (Figure 5A). Conversely, the 6 nM Tl dose reduced *instWUE* by 45.5% and 57.9% in Poblano and Serrano, respectively. In Serrano, the 12 nM Tl dose increased the *instWUE* by 15.7%, with respect to the control (Figure 5B). V increased mean *instWUE* by 70.7% in the Poblano variety, compared to the control (Figure 5C).

### 2.6. Intrinsic Water Use Efficiency (iWUE)

**Figure 6:**
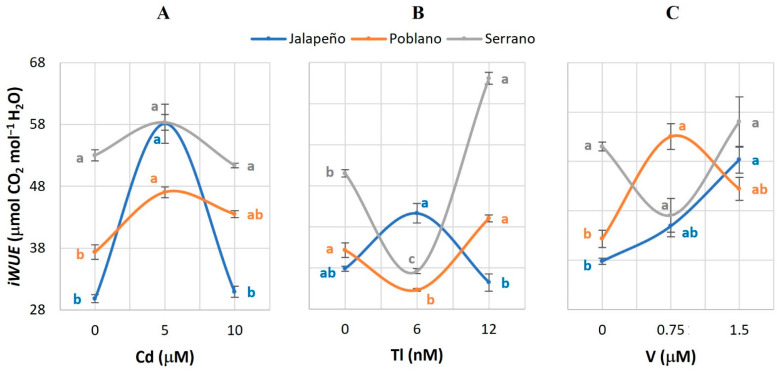
Intrinsic water use efficiency (*iWUE*) in three chili pepper varieties (Jalapeño, Poblano, and Serrano), treated with different doses of cadmium (**A**), thallium (**B**) or vanadium (**C**), for 60 d in a hydroponic system. Means ± SEM. Different letters in each subfigure and variety indicate significant statistical differences among treatments (Tukey, *p* ≤ 0.05). *n* = 4.

Jalapeño and Poblano plants exposed to 5 µM Cd had increases in *iWUE* of 25.9% and 10.1%, respectively, when compared to the control (Figure 6A). In Poblano and Serrano, the addition of 6 nM Tl reduced this variable by 28.2% and 44.9%, compared to the controls. In Serrano, the 12 nM Tl dose increased the *iWUE* by 43.6% with respect to the control (Figure 6B). In Jalapeño, the 1.5 µM V dose increased the *iWUE* value by 68.9%, while in Poblano this increase was 26.3% when applying 0.75 µM V, compared to the control (Figure 6C).

### 2.7. Instantaneous Carboxylation Efficiency (Pn/Ci)

**Figure 7:**
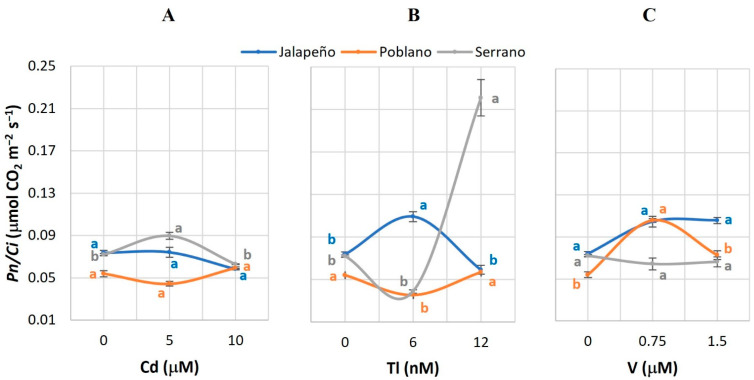
Instantaneous carboxylation efficiency (*Pn*/*Ci*) in three chili pepper varieties (Jalapeño, Poblano, and Serrano), treated with different doses of cadmium (**A**), thallium (**B**) or vanadium (**C**), for 60 d in a hydroponic system. Means ± SEM. Different letters in each subfigure and variety indicate significant statistical differences among treatments (Tukey, *p* ≤ 0.05). *n* = 4.

The *Pn*/*Ci* was increased by 24.7% in the Serrano variety with the addition of 5 µM Cd, compared to the control (Figure 7A). The application of Tl caused significant effects on the *Pn*/*Ci* in the three varieties studied: the 6 nM Tl dose increased it by 48.1% in Jalapeño, and in Poblano it reduced it by 34.3%, in both cases compared to the control. In Serrano, doses of 12 nM Tl tripled the *Pn*/*Ci* compared to the control (Figure 7B). V only had an effect on this variable in the Poblano variety, observing that the 0.75 µM dose increased it by 95.8% compared to the control (Figure 7C).

## 3. Discussion

Together with respiration and transpiration, photosynthesis represents one the three major processes that drive plant growth and development [26].

Alterations in the photosynthetic efficiency of plants after exposure to non-essential metals are due to the modification of various biochemical processes, resulting in dramatic changes in water and ionic relationships that affect electron transport, Calvin-Benson cycle activity, CO_2_ assimilation, pigment content, chloroplast structure, and thylakoid protein composition. However, some non-essential metals in small amounts can produce stimulant effects [27,28], resulting in hormetic dose-response curves.

The accumulation of non-essential metal ions in the cell wall, their absorption through the plasma membrane, and their movement from the cytoplasm to various cell organelles such as vacuoles imply that the plant adjusts its metabolism to achieve homeostasis. This adjustment involves the efficient repair of damaged cell structures during the stress event to which the plant is subjected by exposure to high levels of such metals [29].

Other factors that play critical roles in the regulation of photosynthesis are the concentration and ionic species of the metal, the exposure time, the growth stage of the plant, the tolerance mechanisms evolved, and the ability of each plant genotype to exclude, translocate, and sequester the metal [28]. In other studies, carried out in our research group, we have observed that chili pepper plants display considerable sensitivity to the presence of high levels of non-essential metals in the growth media [30,31,32]. Furthermore, this species exhibits a significant genetic diversity, and therefore, it offers a good biological model to study physiological responses to non-essential metals such as Cd, Tl and V.

Indeed, Cd, Tl and V display particular properties that may influence the responses observed in plants.

Cadmium is a metallic element that, together with Zn and Hg, is part of group 12 of the periodic table, it has an atomic mass of 112.41, atomic number 48, relative density of 8.64 g cm^−3^, radius atomic 1.54 Å. Cadmium is found in nature in low concentration [33,34].

Together with B, Al, Ga and In, Tl is part of group 13 of the periodic table. It is soft, malleable, metallic gray in color, has atomic number 81, relative atomic mass of 204.38, relative density of 11.85 g cm^−3^, with melting and boiling points of 303 °C and 1457 °C, respectively; It has an ionic radius of 1.50 Å, atomic radius of 1.71 Å, it presents two oxidation states Tl^+^ and Tl^3+^, it is similar to cations such as K^+^ [35,36]. 

Vanadium is a transition metal, with a relative density of 6.11 g cm^−3^. In the periodic table it is located as the first transition element of group 5, it has atomic number 23, atomic mass of 50.95, atomic radius of 1.34 Å. It is present in the environment in the oxidation states −3, −1, 0, +2, +3, +4 and +5, the most predominant form being V(+5) [37].

These non-essential metals can alter photosynthesis through the disruption of processes such as stomatal movement, transpiration, and chlorophyll content, among others. One way to study these modifications is through tests with nutrient solutions, which guarantee the absorption of metals by the plant and allow the alterations to be measured with greater precision [38,39,40]. In this study, we established a hydroponic system using the Steiner universal nutrient solution as a source of nutrient supply [41].

### 3.1. Net Photosynthetic Rate (Pn)

The application of Cd in the applied doses did not have significant effects on *Pn* in any of the chili pepper varieties studied (Figure 1A). Contrary to this, decreases in this variable have been reported in Swiss chard (*Beta vulgaris* var. cicla) and mustard (*Brassica juncea* L.) plants exposed to 12 to 24 mg Cd kg^−1^; reductions in chlorophyll synthesis, possibly as a consequence of cell membrane damage, were also observed [42]. Treatments with 10 and 100 µg Cd mL^−1^ decreased *Pn* in carrot *(Daucus carota* L.) [43], while in corn (*Zea mays* L.) and sunflower (*Helianthus annuus* L.) *Pn* was reduced with Cd doses of 10 to 100 µM [44]. Similarly, in cucumber (*Cucumis sativus* L.) plants treated with 50 μM Cd, decreases of 10% in *Pn* were also evident [27]. Alterations in *Pn* can be caused by a decrease in the transport of the electron chain of photosystem II [28].

The treatments with Tl produced positive effects on *Pn* of Serrano chili pepper plants (Figure 1B). In maize and sunflower, the application of 10 to 100 µM Tl reduced *Pn* by 50 and 75% [44], respectively. Furthermore, Tl produced more drastic effects than did Cd, Ni, and Pb. Being isomorphic to K, Tl tends to mimic its movement in guard and companion cells, responsible for stomata opening and closing [45]. This characteristic places Tl as the metal that most inhibits stomatal opening with respect to other metals studied.

The increase in *Pn* in Poblano plants treated with 0.75 µM V in this study (Figure 1C) is in full agreement with that reported in hydroponically grown bell pepper seedlings treated with 5 μM V [31]. Conversely, essential metals such as Zn inhibited *Pn* in common bean (*Phaseolus vulgaris* L.) [46] and in cucumber, the application of 50 μM Cu reduced *Pn* by 20% as compared to the control [27].

The different responses observed in *Pn* among the three chili pepper varieties evaluated can be attributed to the different tolerance mechanisms that each genotype has to deal with the stress caused by exposure to non-essential metals, to their ability to eliminate reactive oxygen species (ROS) efficiently, to the transduction of cellular signals and their biochemical and molecular machinery to sequester and detoxify metal ions [28].

### 3.2. Transpiration Rate (Tr)

The reduction in *Tr* observed in plants of the Jalapeño and Serrano varieties treated with Cd (Figure 2A) has been observed in carrot plants treated with 10 and 100 µg Cd mL^−1^, which is the result of the active interference of Cd in the flow of water through the root towards the other organs of the plant, causing the opening of stomata to be reduced [35]. Similarly, in hybrid poplar plants (*Poplar* spp. hybrids), *Tr* is inhibited by applying Cd, Cu, Cr, and Zn in doses from 5 to 500 mg L^−1^, directly proportional to the concentration of the metals [43].

The positive increases caused by Tl and V in *Tr* in the concentrations studied in the chili pepper varieties (Figure 2B, C) are contradictory to what was reported for essential metals such as Ni, where the application of 0.5 mM NiCl_2_ did not affect *Tr* in rice plants grown in hydroponics [47]. On the other hand, the beneficial element Si, in a 2 mM dose, reduces *Tr* in maize plants grown in hydroponics, due to the alteration in leaf morphology and stomatal density [48].

### 3.3. Stomatal Conductance (Gs)

In Jalapeño and Poblano peppers, the application of Cd reduced the mean value of *Gs* (Figure 3A), which is attributed to the damage caused by Cd in the opening and closing of stomata and to the possible obstruction of the xylem [43]. Furthermore, Cd alters cell turgor as a result of water imbalance and morphological changes in stomata. In spinach (*Spinacia oleracea* L.) and mustard plants exposed to 12 mg Cd kg^−1^ [42] and in carrots treated with 10 and 100 µg Cd mL^−1^, reductions in *Gs* values have also been observed [43]. Under conventional environmental conditions, *Gs* is controlled by guard cell turgor and intercellular CO_2_ concentration [28].

Other non-essential metals also alter the mean values of the *Gs* variable. For example, mercury (Hg) at 50 μmol HgCl_2_ L^−1^ in chili pepper [49], and silicon (Si) at levels of 2 mmol Si L^−1^ in maize plants decrease the values of *Gs* compared to plants not exposed to these elements [48].

Non-essential metals affect photochemical processes in the plant as a consequence of the damage caused in the reaction centers and the alteration of the electron transport chain [50]. The decrease in *Gs* has a negative effect on crop growth and yield [51]. 

In this study, Tl had no effect on the *Gs* of any of the three chili pepper varieties (Figure 3B), presumably because the concentrations of Tl used were low. In an exhaustive search in the scientific literature, no study was found on the effect of Tl on *Gs*.

The increase in *Gs* values caused by V in Serrano pepper represents a beneficial effect of this element on plant biology (Figure 3C). Previous studies have indicated that V has the ability to stimulate physiological processes and increase resistance to abiotic stress when applied at low doses [52]. In bell pepper seedlings, the addition of 5 µM V increased plant growth, induced the development of flower buds and accelerated flowering [31]. Contrarily, in common bean plants treated with 10, 40, and 100 µM Na_3_VO_4_, there are no alterations in *Gs* during the first hours of treatment, but after 12 h the mean values of this variable decrease 50% compared to non-treated plants [53]. The decrease in *Gs* is a direct consequence of the alteration in the content and transport of water by aquaporins and the turgor in the stomatal apparatus [54].

### 3.4. Intercellular CO_2_ Concentration within the Leaf (Ci)

Cadmium had no influence on *Ci* in any of the three chili pepper varieties evaluated (Figure 4A). However, the application of Tl did reduce the mean values of this variable in Serrano chili pepper (Figure 4B). In spruce (*Picea abies* L.) seedlings exposed to metals (1 µM Cd, 0.1 µM Hg, and 60 µM Zn), a decrease in *Ci* was also observed [55]. On the contrary, significant increases in *Ci* were recorded in cucumber plants treated with 50 µM Cu and Cd [22], and in dove-tree (*Davidia involucrata* Baill) exposed to Cd doses of 1 to 30 mg kg^−1^ [56].

The reductions in *Ci* are attributed to the decrease in chlorophyll levels and the partial closure of stomata [55], which occurs to prevent water loss [49], causing the CO_2_ availability to limit the regeneration of the Rubisco enzyme, which ultimately decreases photosynthesis [27].

### 3.5. Instantaneous Water Use Efficiency (instWUE)

The *instWUE* is defined as the amount of carbon fixed in photosynthesis (net CO_2_ input into the leaves) per unit of water transpired (water output from the plant) [51,57]. The effects produced in chili pepper plants exposed to Cd, Tl, and V show that *instWUE* is affected differently in the varieties studied, which may be a consequence of the water expenditure that is occurring in the stomata and the transport of water from the root to the shoot (Figure 5). Metals such as Cd, Cu, and Zn influence water loss through alterations in leaf area, leaf size, leaf blade thickness, reduction of mesophyll intercellular spaces, and reduction of the guard cells [58].

Higher plants can show differential sensitivity to metal ions, which depends on internal factors such as genotype, and external factors such as the type and concentration of the ion. Ionic stress in turn can cause alterations in water absorption and gas exchange [15]. Correct water use efficiency increases the possibility of survival of plants exposed to different types of environmental stress. The repression of the capacity in water use can reduce photosynthetic processes, which restricts the diffusion of CO_2_ due to the closure of stomata, and limits photochemical reactions in the Calvin cycle [57]. In maize plants exposed to a range of 10–100 µM Cd, significant reductions in water absorption values are observed [43], which may result from stomatal closure. Moreover, the reduction in water transport can also result from the degradation of aquaporins, which are the main target of non-essential metals in plants [39].

### 3.6. Intrinsic Water Use Efficiency (iWUE)

The application of Cd, Tl, and V increased the values of the *iWUE* variable in the Jalapeño, Poblano, and Serrano varieties (Figure 6). In sugarcane cv. TCP02-4587 plants subjected to water stress for 30 d, significant increases in *iWUE* were recorded [59]. The *iWUE* is the relationship between net CO_2_ assimilation, stomatal conductance, and water value, and is used to compare photosynthetic properties [60]. The relationship between the reduction of *Gs* and the increase in *Pn* causes the carboxylation capacity to increase as a response to the homeostasis imbalance [59]. Therefore, increases in *iWUE* facilitate carbon absorption under abiotic stress, which implies that increases in *iWUE* stimulate survival and productivity indicators in plants exposed to different types of environmental stress.

### 3.7. Instantaneous Carboxilation Efficiency (Pn/Ci)

In general, increases in *Pn*/*Ci* were observed in plants of the three chili pepper varieties treated with Cd, Tl, and V (Figure 7). These findings coincide with those found in mustard plants grown for 60 d in soil contaminated with approximately 60 mg Cu kg^−1^ and fertilized with biochar [61]. Saline stress causes the same response in various plant species with respect to this variable. Physic nut (*Jatropha curcas* L.) plants cultivated with 100 mmol NaCl L^−1^ for 14 d reduced the mean values of *Pn*/*Ci* [62]. The water deficit decreased the *Pn*/*Ci* by 62 and 25% in sugarcane cv. HoCP93-776 and TCP02-4587 after 30 d, respectively. This variable can be considered as the estimation of the activity of Rubisco, the main enzyme in the carbon fixation process in the Calvin cycle, since its activity is reduced under stress conditions [61].

According to the in-depth search in the scientific literature, there is no previously published information to compare the effects of Cd, Tl and V on the photosynthetic parameters of three chili pepper varieties. However, several studies have documented the individual effects of these elements on plant physiology and metabolism, including photosynthesis [63,64,65].

Photosynthesis is the fundamental process occurring in chloroplasts, which allows plant photosynthetic tissues to convert solar energy into chemical energy, and thus fueling their growth and development [26]. Importantly, this vital process provides over 99% of the energy supply for life on Earth. Therefore, our study contributes to a better understanding of the impact of non-essential elements on photosynthesis, and therefore, on the entire life on the planet.

## 4. Materials and Methods

### 4.1. Experimental Conditions and Plant Material

Chili pepper seeds of the varieties Jalapeño “Emperador” NUN70030, Poblano “Capulín”, and Serrano “Coloso” varieties were germinated in a mixture of peat-moss and perlite (80/20, *v*/*v*) under greenhouse conditions. Once we obtained healthy and vigorous seedlings (60 days after emergence), they were transplanted in black bags with tezontle as a substrate inside a polyethylene greenhouse (19°27′38” N, 98°54′11” W at 2250 m altitude). Plants were acclimated in the greenhouse for 26 d, and during that period they were irrigated with the Steiner nutrient solution at 100% of its original strength [41]. Once the acclimation period was completed, we applied the Cd, Tl and V treatments. The exposure to the metals lasted 60 d. During the experiment, seedlings were grown under a day-length of 12 h, at 28/18 °C day/night temperature, 640 μmol m^−2^ s^−1^ light intensity, and relative humidity of 30% during the day and 86% at night. 

### 4.2. Design of Treatments and Experimental Design

In each of the chili pepper varieties aforementioned and in independent experiments, three concentrations of Cd (0, 5, and 10 µM), three of Tl (0, 6, and 12 nM) and three of V (0, 0.75, and 1.5 µM) were evaluated. The sources of the metals tested were cadmium chloride (CdCl_2_, CAS 10108-64, Sigma Aldrich (Darmstadt, Germany)), thallous acetate (CH_3_COOTl, CAS 563-68-5, Sigma Aldrich), and ammonium metavanadate (NH_4_VO_3_, CAS 7803-55-6, Alfa Aesar (Haverhill, MA, USA)), respectively. The experimental unit consisted of individual plants distributed under a completely randomized experimental design with four replicates per treatment.

To carry out the adequate evaluation of metals, we took into consideration the concentrations of Cd, Tl and V previously studied in other plant species under hydroponic systems [12,66], which did not show phytotoxic effects.

### 4.3. Gas Exchange Measurements

The measurements were made between 11:00 and 14:00 h (the daytime when the highest levels of radiation and temperature are recorded), considering fully expanded mature leaves from the upper part of the plant. The measurements were made on plants that had reached 135 days of age, after 60 days of being exposed to nutrient solutions containing the metals Cd, Tl, or V. The measurements were made with a portable infrared gas analyzer (IRGA, Li-6400^®^, LICOR; Lincoln, NE, USA) with natural light and a CO_2_ concentration in the air between 400 and 420 ppm. The net photosynthetic rate (*Pn*), transpiration rate (*Tr*), stomatal conductance (*Gs*) and intercellular CO_2_ concentration (*Ci*) in the leaf were measured.

With the values of *Pn*, *Tr*, *Gs*, and *Ci*, the following parameters were calculated: (1) the instantaneous water use efficiency (*instWUE*) given by the *Pn*/*Tr* ratio; (2) the intrinsic water use efficiency (*iWUE*) given by the *Pn*/*Gs* ratio; and 3) the instantaneous carboxylation efficiency (*Pn*/*Ci*) according to what was described by [67].

## 5. Statistical Analysis

With the data obtained, an analysis of variance was performed, and the means were compared by the Tukey test (*p* ≤ 0.05) using the SAS software version 9.3 [68].

## 6. Conclusions

Under our experimental conditions, Cd decreased the stomatal conductance and transpiration rate in the three chili pepper varieties evaluated, but increased the instantaneous carboxylation efficiency in Serrano and the instantaneous efficiency in water use in the three varieties. Thallium increased the transpiration rate and the net photosynthesis rate in the three varieties, unbalanced the intercellular concentration of CO_2_ within the leaf and increased the instantaneous carboxylation efficiency and the intrinsic water use efficiency in Serrano and Jalapeño. Vanadium increased most photosynthetic variables in Jalapeño and Serrano and displayed no deleterious effects on the variables measured. 

Photosynthesis represents the basic biological process providing oxygen and food, forming the basis of global food chains, and becoming available most of the energy in the biosphere to living things, thus sustaining virtually all life on Earth. Therefore, a more comprehensive understanding of the effects of non-essential metals such as Cd, Tl and V on this vital process may provide new insights on the mechanisms employed by plants to sense and respond to those environmental cues. Ultimately, photosynthesis is the fundamental factor of crop yield, and thus regulating the photosynthetic process can provide an avenue to keep or even improve yield and quality of crops produced under contaminated environments. The concentrations of these elements in final edible products remain a daunting task and therefore deserve further research.

## Data Availability

The data presented in this study are available on request from the corresponding author.
